# One-year trajectories of mental and physical functioning during and after rehabilitation among individuals with disabilities

**DOI:** 10.1186/s12955-015-0328-z

**Published:** 2015-08-28

**Authors:** Line Preede, Martin Saebu, Paul. B. Perrin, Astrid Nyquist, Haakon Dalen, Erik Bautz-Holter, Cecilie Røe

**Affiliations:** Department of Physical Medicine and Rehabilitation, Faculty of Medicine, University of Oslo, Oslo, Norway; Beitostølen Healthsports Centre, Beitostølen, Norway; Virginia Commonwealth University, Richmond, VA USA; Department of Physical Medicine and Rehabilitation, Ulleval University Hospital, Oslo, Norway

## Abstract

**Purpose:**

First, to evaluate the trajectories of physical and mental functioning in individuals with chronic disabilities receiving adapted physical activity-based rehabilitation. Second, to determine whether demographic factors, disability group, pain, fatigue and self-efficacy at baseline influenced these trajectories.

**Research design:**

A prospective intervention study.

**Methods:**

The study included 214 subjects with chronic disabilities who were admitted to a four-week adapted physical activity-based rehabilitation stay at Beitostølen Healthsports Centre. The subjects completed written questionnaires eight and four weeks before the rehabilitation, at admission to and discharge from the rehabilitation centre and again four weeks and 12 months after discharge. Multilevel models were performed to examine the trajectories of SF-12 physical and mental functioning with possible predictors.

**Results:**

Time yielded a statistically significant effect on physical and mental functioning (*p* < 0.001). Low age (*p* = 0.002), no more than 2 h of personal assistance per week (*p* = 0.023), non-nervous system disability (*p* = 0.019), low pain level (*p* < 0.001) and high chronic disease-efficacy (*p* = 0.007) were associated with higher physical functioning. There was a greater improvement in physical functioning for subjects with lower chronic disease-efficacy at baseline (*p* = 0.036) and with a disability not associated with the nervous system (p = 0.040). Low fatigue (*p* = 0.001) and high chronic disease-efficacy (*p* = 0.004) predicted higher mental functioning. There was also a greater improvement in mental functioning for subjects with high fatigue (*p* =0.003) and low chronic disease efficacy at baseline (p = 0.032).

**Conclusion:**

Individuals with chronic disabilities who participated in an adapted physical activity-based intervention showed statistically significant increases in both physical and mental functioning across the 12 months after the intervention. The greatest improvement was among subjects with a high level of fatigue and low chronic disease-efficacy, as well as disabilities not associated with the nervous system, which has implications for the target groups in future rehabilitation.

## Introduction

Chronic disability is generally defined as the consequence of impairment and a difficulty in functioning at the body, personal, or societal levels in one or more life domains, as experienced by an individual with a health condition in interaction with contextual factors [1]. It may be caused by congenital or acquired diseases or by trauma and other environmental factors [2]. The burden of chronic disability is well recognized [3], and the specific problems vary according to the nature of the impairment. The World Health Organisation (WHO) has defined chronic disability to include moderate to severe health loss. It impacts a person’s well-being and arises from the interaction between health conditions and contextual factors, both personal and environmental [4].

Pain is a subjective experience and the major symptom in musculoskeletal disorders [5, 6]. Pain is closely associated with disability and accounts for the largest reduction in quality of life and functioning [5]. Pain is also a major factor in neurological conditions [7], but fatigue may contribute equally to disability in some conditions [8].

Dobkin et al. (2008) defines fatigue as “a subjective lack of physical and mental energy that interferes with usual activity” [9]. It may be caused by the fact that activities of daily life require most of the individual’s available capacity [10], which might be quite low because chronic disability is associated with a reduced physical activity level [11]. Reduced capacity and exercise form a vicious circle that, together with mobility problems, may result in restricted activities and reduced participation in both work and leisure activities. Eventually, mental and physical functioning is affected [12].

The need for rehabilitation is stressed [13]. However, the effects of rehabilitation on people with disabilities in general, and particularly over the long term, are seldom evaluated. As a result, we have little knowledge about changes and maintenance in functioning over time and possible effective measures of rehabilitation.

The term “adapted physical activity” refers to physical activities adapted to the specific needs of each individual with a disability [14]. Adapted physical activity-based rehabilitations are based on the adaptation of different activities to fit each individual’s needs in the rehabilitation setting. These interventions are in general seldom evaluated, but there are some studies showing the effects of physical activity and environmental factors on physical and mental health and functioning [15–17].

In a previous study conducted at Beitostølen Healthsports Centre (BHC), both physical and mental functioning improved during a four-week adapted physical activity-based rehabilitation [18]. The study lacked long-term follow up and only assessed the outcome at one time point.

Most of the previous studies on chronic disability and rehabilitation outcomes have only one time point for follow up, usually no more than 3 months after discharge from rehabilitation. This study uses longitudinal trajectories to examine paths of variables and how they change over a specific time period. By looking at the trajectories through multilevel modelling (MLM), predictors of individual path changes can be identified. To the authors’ knowledge, none of the previous studies used MLM as recommended for the analysis of longitudinal data [19].

Thus, the main aim of the present work was to evaluate the trajectories of physical and mental functioning over one year in subjects with chronic disabilities who received adapted physical activity-based rehabilitation. Second, we wanted to determine whether demographic factors, type of disability, pain, fatigue and self-efficacy at baseline influenced the trajectories of physical and mental functioning.

## Materials and methods

### Design

The study design was a prospective intervention study.

### Participants and procedures

Subjects with chronic disabilities as defined by WHO, aged 18 years to 73 years (men and woman) and admitted to a four-week rehabilitation stay at BHC were assessed for eligibility. Subjects consenting to participating in and completing the rehabilitation programme were included. Written invitations with information about the study were sent to the participants. Those who accepted the invitation provided written informed consent. The study was approved by the Regional Medical Committee for Research Ethics in Norway (S-08837c 2008/21144). All subjects were examined by a medical doctor upon admission to the rehabilitation centre and by health professionals according to the subject’s specific needs. Physiotherapists, nurses, social workers, and sports rehabilitation specialists comprised the other professions involved. A team was organized for each subject. On the second day, the team and the subject developed a detailed, goal-oriented plan for the rehabilitation.

Between September 2010 and December 2012, data were collected by a written questionnaire administered to the participants eight (baseline) and four weeks before rehabilitation, at admission to and discharge from rehabilitation and again at four weeks and twelve months after discharge (follow-up).

### Rehabilitation programme at BHC

The rehabilitation programme at BHC is based on the vision of adapted physical activity and adapts physical activities to the needs of the individuals [14].

Goal planning is an essential part of the rehabilitation process to enhance subject autonomy, treatment adherence, and feelings of self-efficacy. It provides a basis for individualized treatments through a structured goal-planning process. The subject is an active participant in the rehabilitation process, and the activity of the rehabilitation team is goal oriented and takes into account the preferences of the subject.

The rehabilitation includes social and cultural activities and extensive use of outdoor natural facilities year round. A wide range of services is offered, including adaptation of the environment, technical aids, and individual instruction. The programme is intensive, with 2 to 5 h of physical activity a day, six days a week.

Most of the activities are arranged in groups. The group setting is considered important, encouraging participants to work together, give feedback to each other and exchange activity experiences. During their stay, the participants’ schedules are regularly assessed and adjusted when necessary. The range of activities that the rehabilitation centre offers includes swimming, cross-country skiing, alpine skiing, horseback-riding, aerobics, kayaking and other activities, which allows each individual to determine the activities best suited to him or her.

### Assessments

Demographic data, including age, gender, education, residence, employment, and need for personal assistance, were recorded during an interview with the medical doctor on admission to the rehabilitation centre. Diagnoses were obtained from the referral letter for the rehabilitation stay and were validated by the doctor at admission. The main reasons for disability were grouped according to disorders of the nervous system, disorders of the musculoskeletal system and other disorders.

Perceived physical and mental functioning were measured by the Medical Outcomes Study 12-item Short Form Health Survey (SF-12, licence number QM 027126) [20, 21]. The SF-12 consists of 12 items and yields a Physical Component Summary and Mental Component Summary, which are intended to reflect perceived physical and mental functioning, respectively. The SF-12 has been shown to capture approximately 90 % of the variance in the SF-36 and to reflect the same 8 dimensions [20, 21]. The SF-12 is far less time consuming than the SF-36. It was regarded by the subjects as easier to complete and was chosen to increase the response rate after discharge. The answers were given on a Likert-type scale with 3 or 5 scoring levels for the different items. The Physical and Mental Component Summary (PCS and MCS) norm-based scores for the SF-12 were calculated using the reversed scores of questions 1, 8, 9 and 10 [22]. Mean PCS and MCS for a Norwegian reference population were used for comparison of the study population’s mean scores. The reference scores are 50.3 (SD 8.8) for PCS and 50.6 (SD 9.9) for MCS [20].

The Norwegian versions of three separate scales were used to capture the different elements of self-efficacy. Efficacy for managing chronic disease (Chronic disease-efficacy) was measured by the Self-Efficacy for Managing Chronic Disease 6-Item Scale [23]. A sample item is as follows: “How confident are you that you can keep the fatigue caused by your disease from interfering with the things you want to do?” Responses were given on a 10-point Likert-type scale ranging from *not at all confident* (1) to *totally confident* (10). The scale has been shown to be valid in a sample with 489 subjects with chronic disease and has demonstrated high internal consistency (0.91).

Efficacy for exercise regularly (Exercise-efficacy) was measured by the Exercise Regularly Scale (3-item scale) in the Stanford Chronic Disease Self-Efficacy Scales [24]. A sample item is, “How confident are you that you can do aerobic exercise such as walking, swimming, or bicycling three to four times each week?” Responses were given on a 10-point Likert-type scale ranging from *not at all confident* (1) to *totally confident* (10). The scale has shown good validity in a sample with 478 subjects with chronic disease (the internal consistency was 0.83, and the test-retest reliability was 0.86).

Efficacy for social/recreational activities (Social-efficacy) was measured by the Social/Recreational Activities scale (2-item scale) in the Stanford Chronic Disease Self-Efficacy Scales [24]. A sample item is, “How confident are you that you can continue to do your hobbies and recreation?” Responses were given on a 10-point Likert-type scale ranging from *not at all confident* (1) to *totally confident* (10). The scale has shown to be valid in a sample with 478 subjects with chronic disease (the internal consistency was 0.84, and the test-retest reliability was 0.84).

Pain and fatigue were measured by visual analogue scales (VAS) 100 mm long on a scale of 0–100 (“no pain” to “intolerable pain” and, for fatigue, “not a problem” to “a very big problem”) [25, 26].

### Statistical methods

*T*-test and chi-square statistics were applied to compare the subjects dropping out with those completing the study. Multi-level models (MLMs) were performed to examine whether linear trajectories of the SF-12 physical and mental scores over one year could be predicted by time, sex, age, type of disability, education, employment, personal assistance, pain, fatigue, and self-efficacy. These variables were all entered simultaneously as fixed effects into the models. For the purpose of the analysis, the disability categories were merged into two groups (those with nervous system disabilities and those with other disabilities). The respective mean was subtracted from all variables for the purpose of centring them before being entered into the MLM. SF-12 scores at each of the six time points (baseline, four weeks before admission, at admission to and discharge from rehabilitation, four weeks after discharge, and 12 months after discharge) were entered as the dependent variables in each model. A second set of two MLMs was then run to examine whether any of the statistically significant fixed effects in the first two models interacted significantly with time in the prediction of participants’ physical and mental functioning trajectories, which would indicate that these trajectories changed differentially over time as a function of one of the predictors.

Predictors with significant interactions with time were dichotomized around their mean level (high/low), and paired sample *t*-tests were also conducted to evaluate changes from baseline to the 12-month follow up for subjects with high and low levels of the predictor. All data were analysed using SPSS, version 21. A significance level of 0.05 was adopted.

## Results

### Participants

From the subjects admitted to rehabilitation, 321 were assessed for eligibility and 304 were eligible after exclusion. The exclusion criteria were insufficient knowledge of Norwegian to fill out the questionnaires and severe cognitive disorders. Of the eligible subjects, 246 subjects consented to participation and 32 dropped out before or during the intervention, which resulted in 214 subjects who completed rehabilitation and were included in the study. The gender (*56 %* females) and age (*47 years*) of the 32 subjects who dropped out did not differ significantly from the subjects included in the data analysis (Chi square = 0.000,*p = 0.985 and F = 2.948, p = 0.08*7, respectively). There were no significant differences in the distribution of disability groups between the 32 subjects who dropped out (*50 % nervous system, 31 % musculoskeletal and 19 % others*) and the 214 subjects who completed the programme (see Table 1) (chi square =0.384, *p* = *0.944*). Reported musculoskeletal problems included rheumatic diseases as the most frequent diagnostic entities. Neurological problems included cerebral palsy, multiple sclerosis and inherited motor neuron disorders as the most frequent diagnostic entities. Cerebrovascular diseases, spinal cord injuries and visual impairments were the other reported reasons for disability. The median duration of disease that caused disability was 18.1 years.

**Table 1:** Characteristics of the included subjects

Variables		*n* = 214	%
Age (mean)		51.4	
Gender	Female	119	56
	Male	95	44
Living in town/township (>30 000)		120	56
Education (university level)		95	44
Employed		76	36
Personal assistance (>2 h/week)		42	20
Living alone		74	35
Target group	Nervous system	102	48
	Musculoskeletal	64	30
	Others	48	22

Of the included subjects, 61 did not complete one or more of the 6 questionnaires. They were still included in the MLM, which is robust to missing data. Table 1 shows the characteristics of the included subjects (*n* = 209).

### Trajectory of physical functioning

**Fig. 1:**
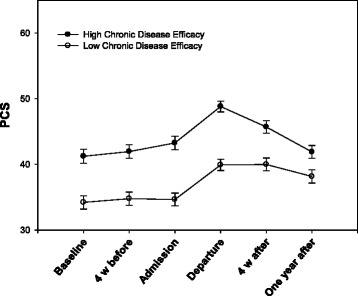
Change in Medical Outcomes Study 12-item Short Form Health Survey Physical Functioning Component Summary (PCS) for the high and low chronic disease-efficacy groups with standard error

**Fig. 2:**
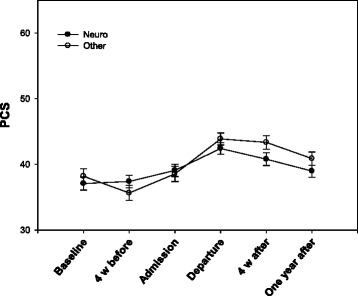
Change in Medical Outcomes Study 12-item Short Form Health Survey Physical Functioning Component Summary (PCS) for the disability groups with standard error

The physical functioning at baseline was rather low, with a mean PCS score of 37.38 (SD 9.60). The MLM showed that physical functioning improved across the six time points (*p* < 0.001) (Table 2), with the main improvement being between admission to and discharge from rehabilitation (Figs. 1 and 2). The mean PCS at discharge was 42.48 (SD 8.16), and at the 12-month follow up, the mean was 39.33 (SD 9.16).

**Table 2:** A hierarchical linear model with time, demographic factors, self-efficacy, fatigue and pain as predictors of Medical Outcomes Study 12-item Short Form Health Survey Physical Functioning Component Summary

Predictor variable	b-weight	SE	df	*t*	*p*-value	95 % Confidence Interval	
						Lower	Upper
Time	0.73	0.10	986.93	7.61	***0.000	0.54	0.91
Sex	0.16	0.92	213.76	0.17	0.862	−1.65	1.97
Age	−0.11	0.04	215.35	−3.16	**0.002	−0.18	−0.04
Employment	2.23	0.98	213.24	2.29	*0.023	0.31	4.16
Living alone	0.03	0.95	214.09	−0.26	0.979	−1.85	1.90
Living in town (>30’)	0.66	0.95	213.31	0.69	0.489	−1.21	2.52
Education	0.10	0.96	212.89	0.10	0.917	−1.79	1.99
Personal assistance (>2 h/week)	−2.26	1.16	215.12	−1.95	0.052	−4.54	0.02
Disability	2.17	0.92	214.16	2.35	*0.019	0.35	3.98
Exercise-efficacy	0.40	0.25	214.22	1.63	0.105	−0.01	0.89
Social-efficacy	0.30	0.22	213.31	1.34	0.180	−0.14	0.74
Chronic disease-efficacy	0.90	0.33	215.64	2.74	**0.007	0.25	1.56
Fatigue	0.00	0.02	213.22	−0.01	0.990	−0.03	0.03
Pain	−0.09	0.02	214.15	−4.35	***0.000	−0.13	−0.05

Note. *= *p* < .05; **= *p* < .01; ***= *p* < .001

A younger age, employment and disability not associated with the nervous system predicted better physical functioning over time (*p* = 0.002, *p* = 0.023 and *p* = 0.019, respectively). Furthermore, subjects with higher chronic disease-efficacy (*p* = 0.007) as well as lower levels of pain (*p* < 0.001) also had better physical functioning (Table 2).

There was a statistically significant interaction between disability and time (*p* = 0.040) and between chronic disease-efficacy and time (*p* = 0.036). The improvement in physical functioning during and after rehabilitation was greater in subjects with a disability not associated with the nervous system and with lower chronic disease-efficacy at baseline (Table 3). After dichotomizing into low and high chronic disease-efficacy around the mean of 6.55, the two groups had the same improvement in physical functioning from baseline to discharge, but the subjects with higher self-efficacy had a greater decline from discharge to the 12-month follow up (Fig. 1). At the 12-month follow up, physical functioning significantly improved compared to baseline, with a mean of 3.16 (SD 7.40) in the low chronic disease-efficacy group (*p* < 0.001) (Table 4). Subjects with high chronic disease-efficacy at baseline showed no statistically significant improvement in physical functioning at the 12-month follow up (mean change 0.63, SD 8.66, *p* = 0.484) (Table 5). Paired comparisons of subjects with or without disabilities associated with the nervous system also showed a significant improvement in both groups from baseline to discharge, but subjects with disabilities not associated with the nervous system did not show as great a decrease as those with a nervous system-associated disability (Fig. 2). At the 12-month follow up, physical functioning significantly improved compared to baseline with a mean of 2.47 (SD 8.50) in the group with disabilities not associated with the nervous system (*p* < 0.006) (Table 6). Subjects with nervous system disabilities showed no statistically significant improvement in physical functioning at the 12-month follow up (mean change 1.26, SD 7.76, *p* = 0.126) (Table 7).

**Table 3:** A hierarchical linear model with statistically significant predictors from Table 2 and their interactions with time as predictors of Medical Outcomes Study 12-item Short Form Health Survey Physical Functioning Component Summary

Predictor variable	b-weight	SE	df	*t*	*p*-value	95 % Confidence Interval	
						Lower	Upper
Time	0.67	0.15	987.89	4.40	***0.000	0.37	0.97
Age	−0.11	0.04	339.34	−2.90	**0.004	−0.19	−0.04
Employment	3.67	1.07	335.55	3.43	**0.001	1.57	5.78
Disability	1.05	1.03	338.45	1.01	0.313	−0.99	3.08
Chronic disease-efficacy	1.60	0.30	339.74	5.36	***0.000	1.01	2.18
Pain	−0.10	0.02	339.80	−4.89	***0.000	−0.14	−0.06
Time *Age	0.00	0.01	989.90	.12	0.906	−0.01	0.02
Time *Employment	−0.38	0.20	985.69	−1.91	0.056	−0.77	0.01
Time *Disability	0.40	0.19	987.44	2.06	*0.040	0.02	0.78
Time *Chronic disease-efficacy	−0.12	0.06	989.67	−2.10	*0.036	−0.23	−0.01
Time *Pain	0.01	0.00	990.81	1.61	0.107	−0.00	0.01

Note. *= *p* < .05; **= *p* < .01; ***= *p* < .001

**Table 4:** Change in Medical Outcomes Study 12-item Short Form Health Survey Physical Functioning Component Summary for subjects with low chronic disease-efficacy

	Mean change	SD	*p*-value	95 % Confidence Interval	
				Lower	Upper
Baseline – Departure (*n* = 102)	5.31	7.60	0.000***	3.82	6.81
Departure – 12 months (*n* = 86)	−2.37	6.85	0.002**	−3.84	−0.91
Baseline – 12 months (*n* = 91)	3.16	7.40	0.000***	1.62	4.70

Note. *= *p* < .05; **= *p* < .01; ***= *p* < .001

**Table 5:** Change in Medical Outcomes Study 12-item Short Form Health Survey Physical Functioning Component Summary for subjects with high chronic disease-efficacy

	Mean change	SD	*p*-value	95 % Confidence Interval	
				Lower	Upper
Baseline – Departure (*n* = 102)	5.31	8.53	0.000***	3.63	6.98
Departure – 12 months (*n* = 91)	−4.90	7.91	0.000***	−6.54	−3.25
Baseline – 12 months (*n* = 94)	0.63	8.66	0.484	−1.15	2.40

Note. *= *p* < .05; **= *p* < .01; ***= *p* < .001

**Table 6:** Change in Medical Outcomes Study 12-item Short Form Health Survey Physical Functioning Component Summary for subjects with disability not associated with the nervous system

	Mean change	SD	*p*-value	95 % Confidence Interval	
				Lower	Upper
Baseline – Departure (*n* = 109)	5.12	8.57	0.000***	3.50	6.75
Departure – 12 months (*n* = 91)	−3.22	7.93	0.000***	−4.87	−1.57
Baseline – 12 months (*n* = 94)	2.47	8.50	0.006**	0.73	4.21

Note. *= *p* < .05; **= *p* < .01; ***= *p* < .001

**Table 7:** Change in Medical Outcomes Study 12-item Short Form Health Survey Physical Functioning Component Summary for subjects with disability associated with the nervous system

	Mean change	SD	*p*-value	95 % Confidence Interval	
				Lower	Upper
Baseline – Departure (*n* = 95)	5.53	7.47	0.000***	4.01	7.05
Departure – 12 months (*n* = 86)	−4.15	7.02	0.000***	−5.66	−2.65
Baseline – 12 months (*n* = 91)	1.26	7.76	0.126	−0.36	2.87

Note. *= *p* < .05; **= *p* < .01; ***= *p* < .001

### Trajectory of mental functioning

**Fig. 3:**
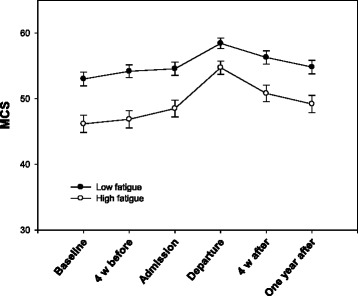
Change in Medical Outcomes Study 12-item Short Form Health Survey Mental Functioning Component Summary (MCS) for subjects with high and low levels of fatigue with standard error

**Fig. 4:**
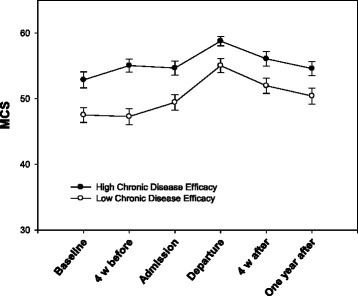
Change in Medical Outcomes Study 12-item Short Form Health Survey Mental Functioning Component Summary (MCS) for the high and low chronic disease-efficacy groups with standard error

Subjects’ baseline values of mental functioning showed a mean MCS score of 49.52 (SD 10.28). The MLM showed that mental functioning improved across the six time points (*p* < 0.001) (Table 8), with the main improvement being between admission to and discharge from rehabilitation (Figs. 3 and 4). The mean MCS at discharge was 56.35 (SD 8.25). At the 12-month follow up, the mean was 52.40 (SD 10.00).

**Table 8:** A hierarchical linear model with time, demographic factors, self-efficacy, fatigue and pain as predictors of Medical Outcomes Study 12-item Short Form Health Survey Mental Functioning Component Summary

Predictor Variable	b-weight	SE	df	*t*	*p*-value	95 % Confidence Interval	
						Lower	Upper
Time	0.85	0.11	986.06	7.39	***0.000	0.62	1.07
Sex	−1.50	1.00	211.55	−1.50	0.135	−3.46	0.47
Age	0.02	0.04	213.42	0.43	0.671	−0.06	0.09
Employment	−0.39	1.06	210.95	−0.37	0.714	−2.49	1.71
Living alone	−1.17	1.03	211.95	−1.14	0.257	−3.21	0.86
Living in town (>30’)	0.79	1.03	211.04	0.77	0.443	−1.24	2.82
Education	0.45	1.04	210.54	0.43	0.667	−1.61	2.51
Personal assistance (>2 h/week)	1.91	1.26	213.15	1.52	0.131	−0.57	4.40
Disability	−0.73	1.00	212.04	−0.73	0.465	−2.71	1.24
Exercise-efficacy	0.41	0.27	212.12	1.52	0.129	−0.12	0.95
Social-efficacy	−0.11	0.24	211.04	−0.44	0.663	−0.59	0.37
Chronic disease-efficacy	1.05	0.36	213.76	2.93	**0.004	0.34	1.76
Fatigue	−0.06	0.02	210.94	−3.22	**0.001	−0.09	−0.02
Pain	−0.04	0.02	212.04	−1.78	0.077	−0.08	0.00

Note. *= *p* < .05; **= *p* < .01; ***= *p* < .001

Subjects with higher chronic disease-efficacy (*p* = 0.004) and lower fatigue (*p* = 0.001) had better mental functioning over time (Table 8).

There was a statistically significant relationship between time and chronic disease efficacy (*p* = 0.032) and between time and fatigue (*p* = 0.003). The improvement in mental functioning during and after rehabilitation was greater in subjects with low levels of chronic disease-efficacy and high levels of fatigue (Table 9). Data were dichotomized into high and low chronic disease-efficacy and fatigue around the means of 6.55 and 49.37, respectively. The group of subjects with high levels of fatigue improved their mental functioning more from baseline to discharge compared to the low-level group, but the decline after discharge was also greater (Fig. 3). Despite the decline, subjects with high levels of fatigue had a significant improvement in mental functioning from baseline to the 12-month follow up (mean difference 3.65, SD 8.46, *p* < 0.001) (Table 10). Subjects with low levels of fatigue had no statistically significant change in mental functioning during the same time period (mean difference 1.29, SD 9.71, *p* = 0.212) (Table 11). Subjects with low chronic disease-efficacy at baseline also improved more in mental functioning from baseline to discharge from rehabilitation (Fig. 4). Although they had a slightly greater decline after discharge than the high chronic disease-efficacy group, subjects with low chronic disease-efficacy had a significant improvement in mental functioning from baseline to the 12-month follow up (mean difference 3.65, SD 9.86, *p* = 0.001) (Table 12). Subjects with high chronic disease-efficacy at baseline showed no statistically significant improvement in mental functioning at the 12-month follow up (mean difference 1.39, SD 8.30, *p* = 0.108) (Table 13).

**Table 9:** A hierarchical linear model with statistically significant predictors from Table 6 and their interactions with time as predictors of Medical Outcomes Study 12-item Short Form Health Survey Mental Functioning Component Summary

Predictor variable	b-weight	SE	df	*t*	*p*-value	95 % Confidence Interval	
						Lower	Upper
Time	0.88	0.11	985.97	7.73	***0.000	0.66	1.10
Chronic disease-efficacy	1.65	0.33	358.49	4.99	***0.000	1.00	2.30
Fatigue	−0.10	0.02	357.72	−5.09	***0.000	−0.14	−0.06
Time * Chronic disease-efficacy	−0.14	0.07	987.18	−2.15	*0.032	−0.27	−0.01
Time * Fatigue	0.01	0.00	985.18	3.00	**0.003	0.00	0.02

Note. * = *p* < .05; ** = *p* < .01; *** = *p* < .001

**Table 10:** Change in Medical Outcomes Study 12-item Short Form Health Survey Mental Functioning Component Summary for subjects with high levels of fatigue

	Mean change	SD	*p*-value	95 % Confidence Interval	
				Lower	Upper
Baseline – Departure (*n* = 114)	8.69	9.13	0.000***	7.00	10.39
Departure – 12 months (*n* = 90)	−5.27	7.85	0.000***	−6.91	−3.62
Baseline – 12 months (*n* = 95)	3.65	8.46	0.000***	1.93	5.37

Note. *= *p* < .05; **= *p* < .01; ***= *p* < .001

**Table 11:** Change in Medical Outcomes Study 12-item Short Form Health Survey Mental Functioning Component Summary for subjects with low levels of fatigue

	Mean change	SD	*p*-value	95 % Confidence Interval	
				Lower	Upper
Baseline – Departure (*n* = 90)	4.35	9.87	0.000***	2.27	6.40
Departure – 12 months (*n* = 87)	−3.37	8.35	0.000***	−5.15	−1.59
Baseline – 12 months (*n* = 90)	1.29	9.71	0.212	−0.75	3.32

Note. *= *p* < .05; **= *p* < .01; ***= *p* < .001

**Table 12:** Change in Medical Outcomes Study 12-item Short Form Health Survey Mental Functioning Component Summary for subjects with low chronic disease-efficacy

	Mean change	SD	*p*-value	95 % Confidence Interval	
				Lower	Upper
Baseline – Departure (*n* = 102)	7.79	10.69	0.000***	5.69	9.89
Departure – 12 months (*n* = 86)	−4.45	8.31	0.000***	−6.23	−2.66
Baseline – 12 months (*n* = 91)	3.65	9.86	0.001**	1.60	5.70

Note. *= *p* < .05; **= *p* < .01; ***= *p* < .001

**Table 13:** Change in Medical Outcomes Study 12-item Short Form Health Survey Mental Functioning Component Summary for subjects with high chronic-disease efficacy

	Mean change	SD	*p*-value	95 % Confidence Interval	
				Lower	Upper
Baseline – Departure (*n* = 102)	5.76	8.49	0.000***	4.09	7.42
Departure – 12 months (*n* = 91)	−4.23	7.99	0.000***	−5.90	−2.57
Baseline – 12 months (*n* = 94)	1.39	8.30	0.108	−0.31	3.09

Note. *= *p* < .05; **= *p* < .01; ***= *p* < .001

## Discussion

The results show that both mental and physical functioning improved during rehabilitation and that improvement remained statistically significant at the 12-month follow up compared to baseline. This supports previous studies indicating an association between rehabilitation and improvement in mental and physical functioning up to three months after rehabilitation [18, 27] and provides new knowledge about the longer-term effects of rehabilitation.

Physical functioning for this sample was low compared to a Norwegian reference population [20]. Previous studies have also found a significant reduction in physical functioning in populations with chronic diseases [12, 28]. Although the sample had a significant improvement in physical functioning from baseline to discharge, physical functioning was still 15 % below the reference population, which is expected considering the nature of the disability in subjects referred to rehabilitation at BHC.

The mental functioning was almost in line with the Norwegian reference population [20]. This is similar to what has been shown in previous research [29, 30]. During the intervention, mental functioning rose to a higher level than the reference population. BHC may be a perfect setting to improve mental functioning for subjects with disabilities, as it is an environment away from everyday life struggles, brings together people who have similar disabilities and health problems, and is guided by well-trained instructors and health workers.

Mental and physical functioning started to improve even before admission to the rehabilitation programme. This might be because of expectations that come from the subjects looking forward to the programme or because they engaged in more exercise to start to improve their functioning before the intervention started. However, the effects of expectations are mainly studied regarding outcome of treatment [31].

In the present study, the improvement in mental and physical functioning from baseline to discharge was more than twice the reported detectable changes of 3 for MCS and 2–3 for PCS [32]. Although the SF-12 is a generic measurement, the clinical significance of changes may vary across disabilities and be influenced by environmental factors. Large variations in clinically important differences have also been reported in the literature [33–36]. Because of the detected change, the improvement in mental and physical functioning from baseline to discharge in this study is of high clinical relevance. With the decline after discharge, the improvements we found in both mental and physical functioning at the 12-month follow up are just below the levels of clinical relevance (2.88 and 1.99, respectively).

The results also show, not surprisingly, that subjects with lower age, those who are employed and those who have disabilities not associated with the nervous system had higher physical functioning over time. Previous findings support the importance of young age in rehabilitation [37–39].

Pain, fatigue and self-efficacy at baseline had effects on the trajectories of physical and mental functioning. Both higher efficacy for managing chronic disease and lower pain predict higher physical functioning at each time point. Higher efficacy for managing chronic disease and lower fatigue predict higher mental functioning at each time point. This result supports the findings of previous studies that investigated the association between self-efficacy and functioning [29, 40, 41] and the association between fatigue and functioning [27, 38].

Subjects with high levels of fatigue at baseline improved their mental functioning, while subjects with low levels did not have any improvement at the 12-month follow up. The biggest improvements happened during the intervention period, where both groups improved. The high-fatigue group degraded more than the low-fatigue group after discharge, but they still showed a significant improvement at the 12-month follow up. It is interesting that the intervention specifically improved the long-term mental functioning of the subjects with high fatigue because it is well known that fatigue impacts a person’s functioning [42–44]. This means that even though the high-fatigue group still had a lower level mental function than the low-fatigue group, the intervention tended to improve it long term.

It is interesting that subjects with lower efficacy for managing chronic disease at baseline had greater improvement in both mental and physical functioning over time than subjects with higher efficacy. This group started their rehabilitation with many insecurities about managing their disease, which might have held them back with regard to improvement. After some time in the BHC environment, it appeared as though they became more secure and observed that others could manage the same disease. This improvement in security might last and help them to maintain their physical and mental functioning after returning to their home environment. It is also important to note that the low-efficacy group maintained their physical functioning without degrading too much after returning to their home environment. This study did not investigate reasons for the maintenance of functioning after discharge, but it is likely that the intervention is a factor. Subjects with high efficacy for managing chronic disease showed no improvement at the 12-month follow up, even though they did improve their mental and physical functioning during the intervention. The fact that subjects with low chronic disease-efficacy at baseline had a greater improvement in functioning was also stated in a previous study on individuals with neuromuscular diseases and multiple sclerosis [45], although that study measured outcome over a shorter time period. These effects could also be the result of statistical regression to the mean over time, whereby participants with a low efficacy at baseline could also be those with the lowest levels of physical and mental functioning and therefore be the groups who have the most room for improvement in functioning during rehabilitation.

### Strengths and limitations

A strength of this study is the use of multi-level modelling, which handles time with unequal spacing and is flexible in handling missing data [19]. This makes it possible to include subjects who did not complete the questionnaire at one or more of the six time points and thereby increases statistical power and improves precision.

Very few studies have evaluated the effects of an adapted physical activity-based intervention. To our knowledge Sprott et al. is one of very few studies that has focused on adapted physical activity for pain patients [46]. Additionally, a study focusing on the effects of equine-assisted activities and therapies for children with cerebral palsy exists [47]. This study contributes important knowledge about the effects of an adapted physical activity-based intervention in a generalized group of subjects with chronic disabilities.

The data collection at admission and discharge occurred at the rehabilitation facility, while data from all other time points were collected in the subjects’ home environment. This might have contributed to bias due to environmental influence. The benefit of subjects completing measures at the facility, and thereby making it a part of the rehabilitation stay, could have decreased the drop-out rate at these time points.

We cannot exclude the possibility of an improvement in physical and mental functioning over time without a rehabilitation stay. However, taking into account that problems the study population face have had a long duration and that there was no improvement during the 8 weeks prior to admission, this improvement seems unlikely. To further investigate the change of improvement, a control group is needed. Because the programme must be provided for those who are in need, it would be ethically challenging to follow a similar group for 12 months without giving them the same intervention during that period.

The subjects who attend rehabilitation at BHC might not fully reflect the Norwegian population with chronic disabilities. It is, of course, a voluntary decision to participate in a rehabilitation stay, and the subjects who chose it might be more motivated to improve their functional skills and physical capacity, as well as to meet new people in such an environment. They also have to be able to leave their everyday environment, family and work situation for a period of 4 weeks to attend rehabilitation.

We have used the word functioning to describe The Medical Outcome Studies Short-Form mental and physical component scores (MCS and PCS). Earlier research has used terms such as mental and physical health, mental and physical functioning and health-related quality of life [18, 48, 49]. The ability of this type of instrument to reflect relevant changes in chronic disabilities has been debated [50], and it has also been debated whether quality of life is a good term regarding the content of these measurements [51]. The present study supports the feasibility of the SF-12 instrument, at least when viewed as a measure of functioning.

This study shows how the trajectories of physical and mental functioning in individuals with disabilities vary over the course of rehabilitation. An adapted physical activity-based intervention is associated with improvements in both physical and mental functioning, and this improvement is statistically significant 12 months after the intervention. An important goal of the rehabilitation programme is sustained long-term improvement. The clinical implication of these results could be that rehabilitation programmes similar to the one at BHC can assess participant self-efficacy and help individuals with disabilities explore the ways in which their self-efficacy influences their engagement in rehabilitation and possibly the resulting gains.

Future research should focus on causes of the decrease in mental and physical functioning after discharge and on trajectories with a longer follow-up period to look for further changes in outcomes. Such knowledge could contribute to improvements in the long-term rehabilitation care for individuals with disabilities.
